# Treatment of Thoracic Outlet Syndrome Complications Assisted with a Cerebral Embolism Protection Device

**DOI:** 10.14797/mdcvj.1103

**Published:** 2022-10-13

**Authors:** Marina Ansuategui-Vicente, Yolanda Tapia-Lopez, Cristina Cases-Perez, Gabriela Ibarra-Sanchez, Ania Garcia Gutierrez, Jose-Antonio Gonzalez-Fajardo

**Affiliations:** 1 Hospital Universitario 12 de Octubre, Madrid, Spain

**Keywords:** cervical rib, retrograde thromboembolization, thoracic outlet syndrome, stroke

## Abstract

We report a case of a stroke and upper limb ischemia in a 27-year-old female secondary to a right cervical rib and retrograde thromboembolization. Follow-up showed complete patency of the vessels after thrombectomy and internal carotid artery stenting followed by transbrachial embolectomy performed with a cerebral protection device. The cervical rib was surgically removed to prevent additional events.

## Background

Thoracic outlet syndrome (TOS) refers to a variety of signs and symptoms caused by compression of the neurovascular bundle by various structures in the area just above the first rib and behind the clavicle, within the confined space of the thoracic outlet. The scalene triangle is the space most commonly involved in TOS, and cervical ribs or anomalous first ribs may compress this space.^1^ TOS can result from a combination of developmental anomalies, injuries, and physical activities that predispose to local compression. Both congenital and acquired thoracic outlet anatomy variants are common and include mainly variations in bone and muscle anatomy.^2^

TOS can involve the brachial plexus, subclavian vein, or subclavian artery although the brachial plexus is the most frequent, responsible for 94% to 96% of cases. Neurological symptoms often present as the initial complaint, with symptoms including pain, paresthesia, or weakness in ulnar distribution involving C8 and T1.^3^ Rare forms of TOS involve the subclavian vein (4-6%) or subclavian artery (1%).^3^ Venous TOS is characterized by significant swelling of the upper extremity, including thrombosis and cyanosis of the upper extremity. Arterial TOS presents as nonradicular pain, pallor, pulselessness, and coolness of the extremity as well as antegrade or retrograde propagation of a thrombus or embolus. This type of TOS is caused by intermittent or prolonged arterial compression of the subclavian artery, typically by a cervical rib. Often, arterial TOS coexists with neurogenic TOS. Signs that point to arterial TOS include unilateral Raynaud-type symptoms of episodic pallor, erythema, and cyanosis.^4^

Cervical ribs are present in 0.5% to 1% of the population and account for 4.5% of thoracic outlet syndrome.^5^ They can be bilateral,^6^ and about 70% of patients with cervical ribs are women.^1^ Arterial TOS accounts for 1% of the cases^7^ and is related to chronic trauma and aneurysm formation.^8^ Compression over time leads to intimal damage, eventual aneurysm formation, thrombosis, and embolic events. It has been described that cervical rib pressure on the right subclavian artery can lead to subclavian thrombosis with right upper extremity embolization and embolic occlusion into the right middle cerebral artery.^9^

We present an unusual cerebral complication and upper limb ischemia caused by TOS and retrograde thromboembolization in a patient with a unilateral first rib treated using an embolic protection device.

## Presentation

A 27-year-old female arrived at the emergency room of a secondary hospital with acute dizziness, language disorder, and weakness of the left upper and lower limb that began 5 hours prior to arrival. Computed tomography (CT) angiography performed on site showed a thrombus in the right middle cerebral artery (MCA). Because she was past the therapeutic window for fibrinolysis, she was urgently brought to our facility for interventional treatment. The CT scan performed in the original center included only the brain, but the supraaortic vessels were not explored. While the patient had no history of neck trauma or cardiovascular risk factors, she reported experiencing Ryanaud’s phenomenon for the last 3 years in both upper limbs.

Her examination on admission revealed a complete right hemispheric syndrome and total left facial and left limb paralysis. A selective angiography through the right femoral artery studied the right carotid artery and confirmed a right middle cerebral artery occlusion. It showed a high-grade stenosis (80%) conditioned by a thrombus adhered to the external and internal right carotid arteries. The stenosis had smooth edges, was well defined, and did not present an underlying vascular lesion, so an embolic origin was suspected.

A mechanical thrombectomy in the MCA was performed to restore blood flow, and a stent in the right cervical internal carotid artery was deployed with good morphological result (the thrombus was not considered for aspiration). The rest of the supraaortic vessels were not explored. Dual antiplatelet therapy was started.

During her first hours in the intensive care unit, the patient neurologically recovered but developed rest pain in the upper right limb. The examination revealed paleness and coolness of the hand with nonpalpable radial, ulnar, brachial, and axillar pulses.

**Figure 1:**
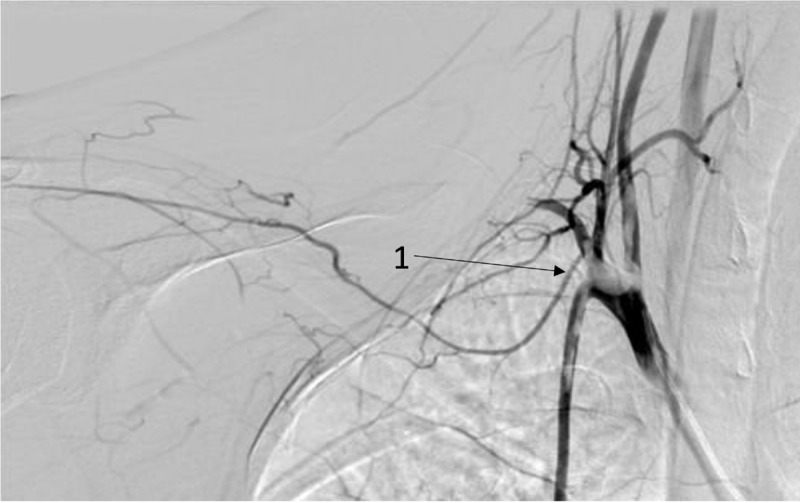
Thrombus in the right subclavian artery, affecting the vertebral and the internal mammary artery.

A nonselective new angiography was performed through the right femoral artery, showing occlusion of the right subclavian artery in its proximal portion that affected the vertebral and the internal mammary artery (Figure 1). Furthermore, a cervical x-ray exposed a cervical right rib. Questioning the patient, she acknowledged a history of pain and paresthesia in the right hand that lasted a few weeks. A duplex ultrasound confirmed the presence of a floating thrombus in the right vertebral artery that extended until the brachial artery. Due to the location of the thrombus, we decided that surgery was necessary.

**Figure 2:**
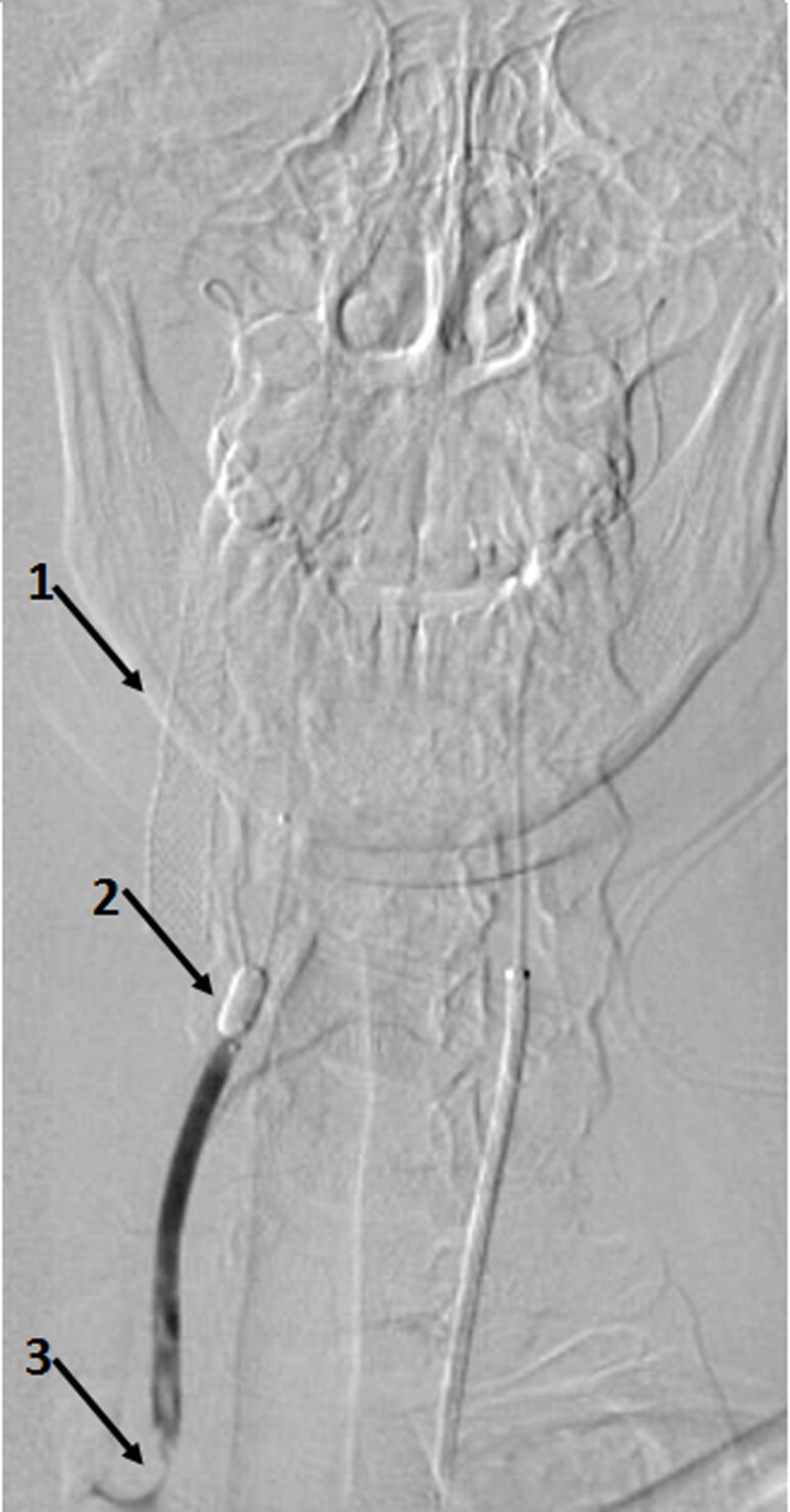
Full view of the approach shows (1) right internal carotid stent, (2) balloon acting as embolic protection device, and (3) remanent thrombus into the proximal vertebral artery.

**Figure 3:**
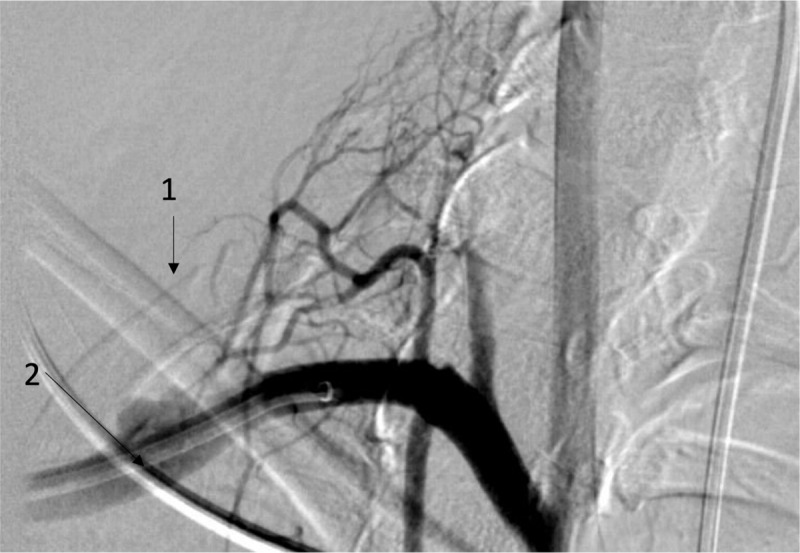
(1) Cervical rib; (2) Sacular aneurysm in the distal subclavian artery.

A transbrachial thrombectomy was carried out with a peculiarity: the left vertebral artery was catheterized and then the right vertebral artery was retrogradely approached (via left vertebral artery basilar artery) with a balloon-tipped microcatheter. Before the thrombectomy, the balloon was opened to avoid thrombi migration to the vertebrobasilar territory. The angiogram showed remanent thrombus into the proximal vertebral artery segment (Figure 2), which was aspired through the brachial artery with a catheter while the balloon was still open. The balloon was then deflated, and the flow was checked without any thrombi migration. Final angiography exposed full patency of the brachial, subclavian, and vertebral arteries with the presence of an aneurysm in the distal subclavian artery (Figure 3). The procedure was well tolerated by the patient, and the right upper limb became warm, showing a radial pulse. A few hours after the surgery, dual antiplatelet therapy was maintained, and anticoagulation was initiated. After 2 days, the patient presented with a ruptured ovarian cyst, and the heparin was suspended. She was dispatched with dual antiplatelet therapy for 1 month and then single antiplatelet treatment with acetylsalicylic acid.

Tumoral markers, initial coagulation tests, autoimmunity tests, and viral serology did not present anomalies. A transesophageal echocardiogram and a Holter monitor eliminated the possibility of a cardioembolic origin. Magnetic resonance angiography ruled out a dissection. We also performed positron emission tomography and computed tomography scans that eliminated the possibility of infections and vasculitis. With all these results, the cervical rib was assumed to be the mechanism of embolization.

Following the study, active elevation of the patient’s arm 130° over her head caused severe damping of the pulse wave in the ultrasound. Seven days after this surgery, she underwent a resection of the right cervical rib using the supraclavicular approach. The aneurysm was repaired through a pericardial patch angioplasty because it was a saccular aneurysm, just above the lesion caused by the rib. The proximal and distal artery was healthy, including the posterior aspect where the patch was sutured. We did not consider bypass necessary.

The patient was dispatched the day after surgery and was completely asymptomatic. No further neurologic or ischemic events were reported at the 15-month follow-up.

## Discussion

Cervical ribs are a common cause of arterial (subclavian) TOS. This pathology can lead to three major events: partial or complete thrombosis of the subclavian artery, poststenotic dilatation of the subclavian artery, or poststenotic subclavian aneurysm (as was our case).^10^ All these entities may cause distal embolization or acute upper limb ischemia and uncommonly retrograde thrombus propagation from the subclavian artery to vertebral, carotid, or cerebral territory. Both retrograde and antegrade thromboembolism may occur.

The reported incidence of cervical ribs in the general population is about 1%.^5^ They are found twice as frequently in females (68%) than in males (32%) and are bilateral in greater than 50% of involved cases. Whereas cervical ribs are asymptomatic in 90% of cases, symptomatic cervical ribs typically result in neurogenic TOS and, less commonly, arterial TOS.^2^

There are limited reported cases of retrograde thromboembolism from subclavian artery TOS causing a cerebral ischemic event.^8,11,12,13^ The reported stroke treatment in these cases was fibrinolysis or conservative management with anticoagulation. In fact, in one of the cases, surgical rib resection was postponed to avoid cerebral embolization.^12^

In our case, we had the means to perform a mechanical thrombectomy and, because the limb vascularization was compromised, we also carried out a transbrachial thrombectomy. What makes this case special is the use of an embolic protection device. Distal occlusion devices usually include a balloon catheter to temporarily stop blood flow in a vessel while the intervention is performed. Any residual thrombi can be removed through an aspiration catheter prior to the distal occlusion being released, which we did in our case. Since our patient was 27 years old, we thought that it was important to assure vertebral and cerebral patency. This kind of treatment has not been reported in the literature before.

To clarify, the first CT did not include the supraaortic vessels. Following the hospital’s protocol, the patient was referred to our facility for stroke treatment and directly underwent mechanical thrombectomy. The right subclavian artery was not studied during the stroke treatment.

Although an embolic event leading to a cerebrovascular accident is rare in patients with arterial TOS, these patients may have previous upper limb symptoms. In our case, the patient presented with pain and paresthesia of the right hand 2 to 3 weeks before the major event. We highlight the importance of ruling out a cervical rib in young patients with these symptoms.

## Conclusion

Although cerebral events due to arterial TOS are unusual, the consequences could be fatal when they occur. We report a new application of a well-known technique that may help protect the distal vessels in this sensitive territory.

## References

[1] Sanders RJ, Hammond SL, Rao NM. Diagnosis of thoracic outlet syndrome. J Vasc Surg. 2007 Sep;46(3):601-4. doi: 10.1016/j.jvs.2007.04.050 17826254

[2] Masocatto NO, Da-Matta T, Prozzo TG, Couto WJ, Porfirio G. Thoracic outlet syndrome: a narrative review. Rev Col Bras Cir 2019 Dec 20;46(5):e20192243. doi: 10.1590/0100-6991e-20192243/31859722

[3] Urschel HC, Kourlis H. Thoracic Outlet Syndrome: a 50-year experience at Baylor University Medical Center. Proc (Bayl Univ Med Cent). 2007 Apr;20(2):125-35. doi: 10.1080/08998280.2007.11928267 17431445 PMC1849872

[4] Kuhn JE, Lebus V GF, Bible JE. Thoracic outlet syndrome. J Am Acad Orthop Surg. 2015 Apr;23(4):222-32. doi: 10.5435/JAAOS-D-13-00215 25808686

[5] StatPearls [Internet]. Bethesda, MD: National Library of Medicine; c2022. Fliegel BE, Menezes RG. Anatomy, Thorax, Cervical Rib; 2021 Jul 26 [cited 2022 Sep 2]. Available from: https://www.ncbi.nlm.nih.gov/books/NBK541001/

[6] Pollak EW. Surgical anatomy of the thoracic outlet syndrome. Surg Gynecol Obstet. 1980 Jan;150(1):97-103. PMID: 7350712 7350712

[7] Hood DB, Kuehne J, Yellin AE, Weaver FA. Vascular complications of thoracic outlet syndrome. Am Surg. 1997 Oct;63(10):913-7. PMID: 9322672 9322672

[8] Sharma S, Kumar S, Joseph L, Singhal V. Cervical rib with stroke as the initial presentation. Neurol India. 2010 Jul-Aug;58(4):645-7. doi: 10.4103/0028-3886.68691 20739814

[9] Purves-Stewart J, Symonds CP. Proceedings of the Section of Neurology of the Royal Society of Medicine Two Cases of Thrombosis of Subclavian Artery, with Contralateral Hemiplegia of Sudden Onset, probably Embolic. Brain. 1927 Jun 1;50(2):259-60. 10.1177/003591572702000816 PMC2100561 19985906

[10] Davidovic LB, Kostic DM, Jakovljevic NS, Kuzmanovic IL, Simic TM. Vascular Thoracic Outlet Syndrome. World J Surg. 2003 May;27(5):545-50. doi: 10.1007/s00268-003-6808-z 12715220

[11] Lee TS, Hines GL. Cerebral embolic stroke and arm ischemia in a teenager with arterial thoracic outlet syndrome: a case report. Vasc Endovascular Surg. 2007 Jun-Jul;41(3):254-7. doi: 10.1177/1538574407299780 17595394

[12] Jusufovic M, Sandset EC, Popperud TH, Solberg S, Ringstad G, Kerty E. An unusual case of the syndrome of cervical rib with subclavian artery thrombosis and cerebellar and cerebral infarctions. BMC Neurol. 2012 Jun 28;12:48. doi: 10.1186/1471-2377-12-48 22741548 PMC3475120

[13] Hafeezullah S, Raghav J, Anuradha N. An interesting case of young stroke. World J Med Sci. 2014;10(3):275-8.

